# Exploring the adverse effect of fine particulate matter (PM_2.5_) on wildland firefighters’ pulmonary function and DNA damage

**DOI:** 10.1038/s41598-024-58721-4

**Published:** 2024-04-04

**Authors:** Jinjuta Panumasvivat, Ratana Sapbamrer, Nalin Sittitoon, Supakit Khacha-ananda, Wuttipat Kiratipaisarl, Wachiranun Sirikul, Wittawat Insian, Pheerasak Assavanopakun

**Affiliations:** 1 Department of Community Medicine, Faculty of Medicine, Chiang Mai University, Chiang Mai, 50200 Thailand; 2 Environmental and Occupational Medicine Excellence Center (EnOMEC), Department of Community Medicine, Faculty of Medicine, Chiang Mai University, Chiang Mai, 50200 Thailand; 3 School of Environmental Health, Institute of Public Health, Suranaree University of Technology, Nakhon Ratchasima, 30000 Thailand; 4 Department of Forensic Medicine, Faculty of Medicine, Chiang Mai University, Chiang Mai, 50200 Thailand; 5 Center of Data Analytics and Knowledge Synthesis for Health Care, Chiang Mai University, Chiang Mai, 50200 Thailand; 6 Office of Disease Prevention and Control 1 Chiang Mai, Department of Disease Control, Ministry of Public Health, Chiang Mai, 50000 Thailand

**Keywords:** Wildland firefighters, Particulate matter, Pulmonary function, DNA damage, Environmental sciences, Biomarkers

## Abstract

Chiang Mai encounters severe pollution during the wildfire season. Wildland firefighters encounter various hazards while engaged in fire suppression operations, which encompass significant exposure to elevated concentrations of air pollutants resulting from combustion, especially particulate matter. The adverse effects of wildfire smoke on respiratory health are a significant concern. The objective of this study was to examine the potential adverse effects of PM_2.5_ exposure on the respiratory function and DNA damage of wildland firefighters. This prospective cohort study conducted in Chiang Mai from January to May 2022 planned to evaluate the health status of wildland firefighters during the pre-peak, peak, and post-peak ambient air pollution seasons. The measurement of PM2.5 was done at every forest fire station, as well as utilizing data from the Pollution Control Department. Participants received general health examinations, spirometry evaluations, and blood tests for DNA damage analysis. Pair *t*-tests and multiple regression models were used to examine the connection between pulmonary function parameters (FVC, FEV_1_) and PM_2.5_ concentration, with a significance level of P < 0.05. Thirty-three peak-season and twenty-one post-peak-season participants were enrolled. Four pre-peak-season wildland firefighters had FVC and FEV_1_ declines of > 15%. Multiple regression analysis showed a negative association between PM_2.5_ exposure and FVC% predicted (− 2.81%, 95% CI − 5.27 to − 0.34%, P = 0.027) and a marginally significant negative correlation with FVC (− 114.38 ml, 95% CI − 230.36 to 1.59 ml, P = 0.053). The remaining pulmonary measures showed a statistically insignificant decline. There were no significant changes in DNA damage detected. Wildland firefighters suffered a significant decline in pulmonary function associated with PM_2.5_ exposure. Spirometry is crucial for monitoring and promptly identifying respiratory issues that occur during wildfire seasons. Further research is recommended to explore DNA damage alterations and their potential association with PM2.5.

## Introduction

Ambient air pollution is a continually expanding global health concern. The World Health Organization (WHO) estimated that 99% of the global population breathes air that exceeds WHO guidelines, resulting in 4.2 million deaths each year due to ambient air pollution^1^. This issue has been significantly impacted by climate change and regionally distinct weather events^2^. Moreover, specific sources, such as smoke from wildfires, can contribute to ambient air pollution. In 2019, Global Forest Watch recorded more than 4.5 million fires worldwide, either directly or indirectly caused by climate change^3^. Wildland fire emissions contain a range of air pollutants, such as fine and coarse particulate matter (PM), gases, carbon monoxide (CO), methane, nitrous oxide (N_2_O), nitrogen oxides (NOx), volatile organic carbon (VOC), and many other air toxics. Therefore, it can lead to numerous adverse health consequences in various human systems^4,5^. There is evidence of a significant association between exposure to conflagration smoke or PM_2.5_ and all-cause mortality and respiratory morbidity^6^. Regarding the respiratory system, it was discovered that some lung function parameters declined due to wildfires^5,7,8^. In areas where wildfires have a greater impact, the issue of understanding the effects of wildfires is ongoing and must be addressed with greater awareness.

Firefighters are particularly at risk for wildfire hazards due to their exposure to extreme temperatures, hazardous chemicals and substances, shift work, and psychological stress in emergency fire suppression^9^. Although wildland firefighters (WLFF) are required to wear personal protective equipment (PPE), it is unlikely that they would use self-contained breathing apparatus (SCBA) like urban firefighters^10,11^. Due to the excessive weight and limited duration of use of SCBA, WLFF are at a higher risk of inhaling hazardous substances^12^. In addition to differences in working conditions, while few PM toxicity studies focus on wildfire sources, those that do suggest that finer particles are more adverse than coarser particles and that wildfire PM may be more toxic than urban PM^13^. One main system affected is the respiratory system. Changes in pulmonary function were reported regardless of smoking history^14^. Pulmonary inflammation is the established mechanism of change, leading to the formation of radicals^15^. Conducting research on either pulmonary function or DNA damage indicators would provide us with a comprehensive understanding.

Chiang Mai is one of the world's notable places with the most severe levels of ambient air pollution, surpassing the daily standard set by the air quality index. This is particularly evident during the peak of the annual wildfire season, which typically occurs from February to April. During the seasonal haze, PM_2.5_ levels in Chiang Mai exceeded the WHO annual air quality guideline value by four times or more. The previous study showed that one of the primary sources of ambient air pollution in Chiang Mai affects the biomass community with burning from forest fires and agricultural burning^16^. Forest fires in Chiang Mai doubled, increasing from 1537 occurrences in fiscal year 2022 to 3689 occurrences in fiscal year 2023^17^. Currently, there is governmental involvement that wants to support and maintain wildland firefighters’ health but needs more information to assess fit-to-work and surveillance of wildland firefighters’ health, which might be established as a national standard. Our previous study revealed that WLFF in Thailand lacked sufficient medical surveillance and fitness-for-duty assessments^18^. Therefore, it was crucial to examine the negative health effects and implement a suitable occupational health and safety program for WLFF. To bridge this knowledge gap, we aimed to investigate the potential adverse effects of PM_2.5_ exposure on the respiratory function and DNA damage of WLFF during two phases: the pre-to-peak season and pre-to-post-peak season. Spirometry and DNA damage measures were utilized to describe both cell and clinical levels since research revealed that wildfire can induce deleterious effects from cell to clinical presentation.

## Materials and methods

### Study design and population

**Figure 1:**
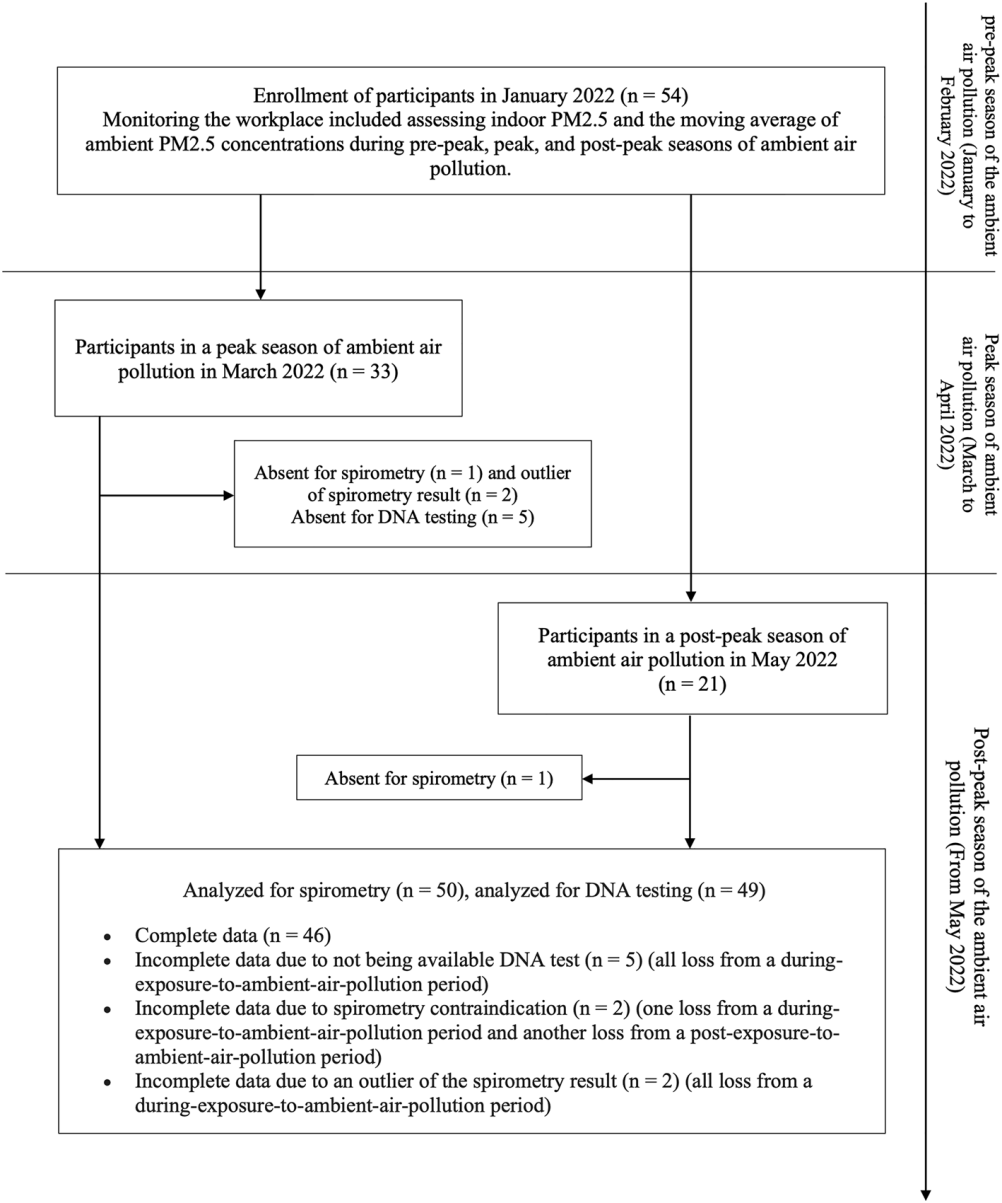
The study flow diagram.

This prospective cohort study was conducted from January 2022 to May 2022, starting from a pre-peak season in January 2022 to a peak season of ambient air pollution in March 2022 and a post-peak season of ambient air pollution in May 2022. According to a previous study^18^ that recruited from all stations in Chiang Mai province administered by the Organization of Protected Areas, Regional Office 16, Department of National Parks, Wildlife, and Plant Conservation, Thailand, seven stations volunteered to participate in our study. The study's inclusion criteria required that participants had a minimum of 1 year of work experience and be at least 18 years of age. The study excluded participants who were no longer accessible for follow-up during the study period. A total of fifty-four wildland firefighters were recruited during the pre-peak season of ambient air pollution, which commenced in January 2022 and served as the baseline. In the final cohort observation, a total of thirty-two participants were observed during the peak season, while twenty participants were observed during the post-peak season of ambient air pollution. Figure 1 represented the study flow and timeline of participants’ surveillance during both periods.

### Data collection for working-area monitoring

The primary focus of participants' professional responsibilities revolved around the national park region. The likelihood of this region undergoing a transformation into large-scale wildfires was substantial. Their responsibility entailed being present at the station and constantly ready to participate in the suppression of wildfires in the event of any reported areas of high fire activity. Furthermore, their responsibilities encompassed the construction of firebreaks within the high-risk region of the hotspot during the pre-peak season. For the working area sampling, primary data from ambient work assessments was utilized at each forest fire station. Additionally, secondary data on the ambient air index was obtained from the Pollution Control Department, Ministry of Natural Resources and Environment, Thailand (PCD). The indoor PM_2.5_ assessment at the station was measured using the real-time optical scattering method by Dusttrak II Aerosol Monitors for an entire eight-hour workday. This method was recommended by the Thai regulations (announcement of the Department of Health on the monitoring of air quality in public buildings, B.E. 2565). The indoor PM_2.5_ concentration was presented as an eight-hour mean average. For ambient PM_2.5_ data, PCD continuously monitored it using beta-ray attenuation measurements. This information was computed and presented as a 3-day moving average.

### Data collection for health assessments

The health assessments consisted of general health information, spirometry evaluations, and blood test for DNA damage analysis. Participants' general information includes their age, gender, body weight (kg), height (cm), waist circumference (cm), body mass index (BMI, kg/m^2^), smoking status, and vital signs. BMI was categorized into four groups by Asian BMI classification: underweight (< 18.5 kg/m^2^), normal weight (18.5–2.9 kg/m^2^), overweight (23–24.9 kg/m^2^), and obese (≥ 25 kg/m^2^)^19^. Participants' vital signs, including blood pressure and pulse rate, will be recorded to determine if they were able to do spirometry. Six milliliters of venous blood samples were collected twice from each subject.

Blood samples were transferred to be centrifuged at 3000 revolutions per minute (rpm) for fifteen minutes to enable the collection of plasma. DNA damage was measured using the alkali comet assay based on the method published by Singh et al.^20^ (Supplementary Information). Centrifugation was used to isolate lymphocytes, which were then prepared for electrophoresis and analyzed using a fluorescence microscope equipped with an imaging system and COMET Assay software. Tail length (the distance of DNA migration) and tail moment (the distance between the center of gravity of the head and the center of gravity of the tail) were the two main parameters measured. These values were measured and displayed in microns (μm).

The SpiroMaster PC-10 was used to evaluate the spirometry test. The procedures was conducted using recommendation standardization of spirometry from ATS/ERS^21^. Participants required to completed at least three acceptable graphs in accordance with ATS/ERS requirements^21^. Collecting parameters including FEV_1_, FCV, FEF_50_, and FEV_1_/FCV. The Thai Siriraj equation was used as a reference for predicting the value^22^. Interpretation of pulmonary function was calculated by using fixed ratio criteria to determine normal lung function, obstructive lung, restrictive lung, and mixed abnormal. The determination of abnormally reduced lung function was determined by using a 10% decline in FEV_1_ from baseline, and a significant reduction was considered when measured FEV_1_ declined by 15%^23^.

### Statistical analysis

The mean of ambient PM_2.5_ concentration was calculated as the 3-day moving average 14 days before the test. The indoor PM_2.5_ was presented as mean average. In this study, paired *t*-test was used to compare changes in pulmonary function test and multiple regression statistical models was used to determine the correlation between lung function indices (FVC, FEV_1_) and PM_2.5_ concentration (0 day). Multiple imputation was used to assume the incomplete data for missing data in TL and TM before using multivariate regression based to compare difference of TL an TM between the two periods. Statistical analyzes were performed using STATA software (version 16.0) and statistical significance for P-values < 0.05 were considered.

### Ethical considerations

This study and the protocol were approved by the Research Ethics Committee, Faculty of Medicine, Chiang Mai University, Thailand (Study code: COM-2564-08308).

### Institutional review board statement

This study was conducted following the Declaration of Helsinki, and the protocol was approved by the Research Ethics Committee, Faculty of Medicine, Chiang Mai University, Thailand (Study code: COM-2564-08308).

### Informed consent statement

Informed consent was obtained from all subjects involved in the study.

## Results

### Baseline characteristics of wildland firefighters and difference between two groups

All 54 participants actively participated in this study until its conclusion. Nevertheless, only 50 of the 54 wildland firefighters with completed spirometry data and 49 with completed DNA samples were available for analysis. Most of the participants were men, and their average age was 41.81 years. Following the phase of follow-up, we categorized all participants into two categories.

In the pre-to-peak season group, the mean age of participants was 41.59 + 10.19 years. In the pre-to-post-peak season group, the mean age of participants was 42 + 9.99 years. The majority of participants in both groups were male, engaged in smoking, and did not have any pre-existing medical conditions. There were no statistically significant differences observed in the characteristics of the two groups. Table 1 provided the pre-peak and pre-post-season groups' baseline characteristics.

**Table 1:** Baseline characteristics of wildland firefighters and characteristics between pre-to-peak season and pre-to-post-peak season group.

Characteristics	Total (N = 54)	Pre-to-peak season of ambient air pollution (n = 33)	Pre-to-post-peak season of ambient air pollution (n = 21)	P-value
Age (yrs.), mean ± SD	41.81 ± 10.02	41.59 ± 10.19	42 ± 9.99	0.879
Weight (kg), mean ± SD	67.43 ± 9.85	66.11 ± 8.71	69.62 ± 11.17	0.063
Height (cm), mean ± SD	166.76 ± 6.76	165.89 ± 5.43	168.10 ± 8.35	0.100
BMI (kg/m^2^), mean ± SD	24.16 ± 3.12	23.92 ± 3.14	24.59 ± 3.09	0.251
Gender (n, %)				
Male	52 (96.30)	32 (96.97)	20 (95.24)	0.642
Female	2 (3.70)	1 (3.03)	1 (4.76)	
Smoking status (n, %)				
Non-smoker	13 (24.07)	8 (24.24)	6 (28.57)	0.928
Ex-smoker	14 (25.93)	8 (24.24)	5 (23.81)	
Active smoker	27 (50.00)	17 (51.52)	10 (47.62)	
Underlying diseases				
No	41 (75.93)	24 (72.73)	17 (80.95)	0.475
Yes	13 (24.07)	9 (27.27)	4 (19.05)	
Allergic rhinitis	3 (5.56)	2 (6.06)	1 (4.76)	
Asthma	4 (7.41)	3 (9.09)	1 (4.76)	
Cardiac arrythmia	2 (3.70)	2 (6.06)	0	
Cardiovascular disease	2 (3.70)	2 (6.06)	0	
Hypertension	5 (9.26)	3 (9.09)	2 (9.52)	

*BMI* body mass index, *cm* centimeters, *kg* kilograms, *SD* standard deviation.

### Ambient and indoor PM_2.5_ concentration between pre-peak, peak, and post-peak season

**Figure 2:**
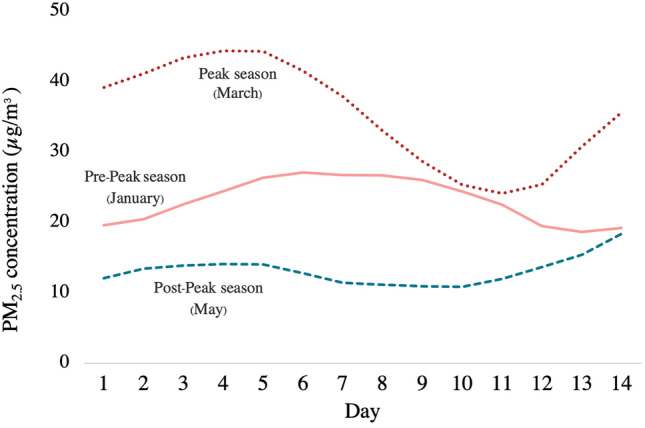
Comparison of the 3-day moving average concentration of ambient PM_2.5_ (14 days before the data collection date between the pre-peak season of ambient air pollution (January), the peak season of ambient air pollution (March), and post-peak season of ambient air pollution (May)).

The ambient air monitoring was used to represent the average exposure of wildland firefighters operating at the scene during each period. Figure 2 showed the 3-day moving average of ambient PM_2.5_ prior to 14 days of data collection for each phase. In the pre-peak, peak, and post-peak seasons, ambient PM_2.5_ concentrations were 23.1 ± 3.2, 35.3 ± 7.4, and 13.1 ± 2.0 g/m^3^, respectively. The average concentration of indoor PM_2.5_ ranged between 17 and 24.44 g/m^3^ during the “pre-to-peak season” and between 10.78 and 85.95 g/m^3^ during the “pre-to-post-peak season.” Table 2 showed the “pre-peak season” and “post-peak season” mean indoor PM_2.5_ concentrations for each station. In every location, peak season PM_2.5_ concentrations were significantly higher than pre-peak season concentrations, and post-peak season PM_2.5_ concentrations were significantly lower than pre-peak season concentrations.

**Table 2:** Mean average indoor PM_2.5_ concentration (µg/m^3^) between pre-to-peak season (January to March) and pre-to-post-peak season (January to May).

Area	Pre-peak season of ambient air pollution	Peak season of ambient air pollution	P-value
Pre-to-peak season (January to March)			
Area 1: Chom Thong	42.26 ± 1.01	62.34 ± 8.53	< 0.001
Area 2: Phu Ping	78.82 ± 28.55	95.82 ± 18.89	< 0.001
Area 4: Sri Lanna	43.89 ± 20.00	68.33 ± 45.55	< 0.001
Area 5: CMFFOC	43.72 ± 19.41	61.23 ± 24.85	< 0.001
Pre-to-post-peak season (January to May)			
Area 3: Mae Takhrai	121.90 ± 28.88	35.95 ± 2.85	< 0.001
Area 6: Huay Hong Krai	67.60 ± 14.26	25.08 ± 5.32	< 0.001
Area 7: Ob Khan	38.56 ± 6.09	27.78 ± 5.29	< 0.001

### Pulmonary function between two groups

This study found a significant difference in observed FVC, percent predicted FVC, observed FEV_1_, and percent predicted FEV_1_ among participants in the pre-to-peak season group (P < 0.05). The mean differences between observed FVC and observed FEV_1_ in pre-peak season were − 0.28 + 0.59 L (95% CI − 0.50 to − 0.06, P = 0.013) and − 0.21 + 0.49 L (95% CI − 0.39 to − 0.03, P = 0.025), respectively. For the pre-to-post-peak season, it was no significant difference in pulmonary function between the two periods. The overall details are shown in Table 3.

**Table 3:** Mean average and mean changes in pulmonary function among pre-to-peak and pre-to-post-peak season periods.

Parameters	Pre-peak season of ambient air pollution	Peak season of ambient air pollution	Mean changes	95% CI	P-value*
Pre-to-peak season (January to March) n = 30					
FVC, observed) (L)	4.13 ± 0.85	3.84 ± 0.77	− 0.28 ± 0.59	− 0.50, − 0.06	**0.013**
FVC, percent predicted (%)	110.05 ± 17.79	102.39 ± 13.31	− 7.86 ± 15.852	− 13.78, − 1.94	**< 0.001**
FEV_1_, observed) (L)	3.35 ± 0.71	3.14 ± 0.65	− 0.21 ± 0.49	− 0.39, − 0.03	**0.025**
FEV_1_, percent predicted (%)	107.69 ± 15.83	102.01 ± 12.75	− 5.82 ± 13.63	− 10.91, 0.73	**0.002**
FEV_1_/FVC (%)	81.32 ± 5.99	81.90 ± 6.20	0.58 ± 3.64	− 0.78, 1.94	0.388
FEF_50_, observed (L)	4.61 ± 1.68	4.33 ± 1.56	− 0.28 ± 1.15	− 0.70, 0.15	0.201
Pre-to-post-peak season (January to May) n = 20					
FVC, observed) (L)	4.00 ± 0.56	4.02 ± 0.65	0.10 ± 0.24	− 0.02, 0.21	0.090
FVC, percent predicted (%)	103.58 ± 12.74	103.83 ± 14.42	0.25 ± 8.33	− 3.65, 4.15	0.849
FEV_1_, observed (L)	3.31 ± 0.53	3.38 ± 0.53	0.06 ± 0.17	− 0.02, 0.14	0.113
FEV_1_, percent predicted (%)	105.38 ± 13.79	105.54 ± 14.35	0.16 ± 6.92	− 3.07, 3.40	0.879
FEV_1_/FVC (%)	82.85 ± 5.11	82.77 ± 5.64	− 0.08 ± 2.11	− 1.07, 0.90	0.865
FEF_50_, observed (L)	4.82 ± 1.67	4.72 ± 1.51	− 0.10 ± 0.59	− 0.38, 0.17	0.454

*Paired *t*-test. *FVC* forced vital capacity, *FEF*_*50*_ forced expiratory flow at 50% of the forced vital capacity, *FEV*_*1*_ forced expiratory volume in 1 s, *L* liters.

Significant values are in bold.

Out of the 30 individuals in the pre-to-peak group, seven of them, with six having normal lung function and one having obstructive lung function, experienced a significant decline in volume on the pulmonary function test. Four individuals observed a decrease in lung function exceeding 15%. In addition, the lung function of one participant shifted from normal to obstructive.

Among the group of 20 individuals observed during the pre-to-post-peak season, a significant decline in lung function was observed. Two individuals with abnormal lung function had a recovery to normal lung function, one due to obstruction and the other due to restriction. One participant underwent a shift from a normal lung to a restrictive lung without reporting a significant decrease in lung function.

**Figure 3:**
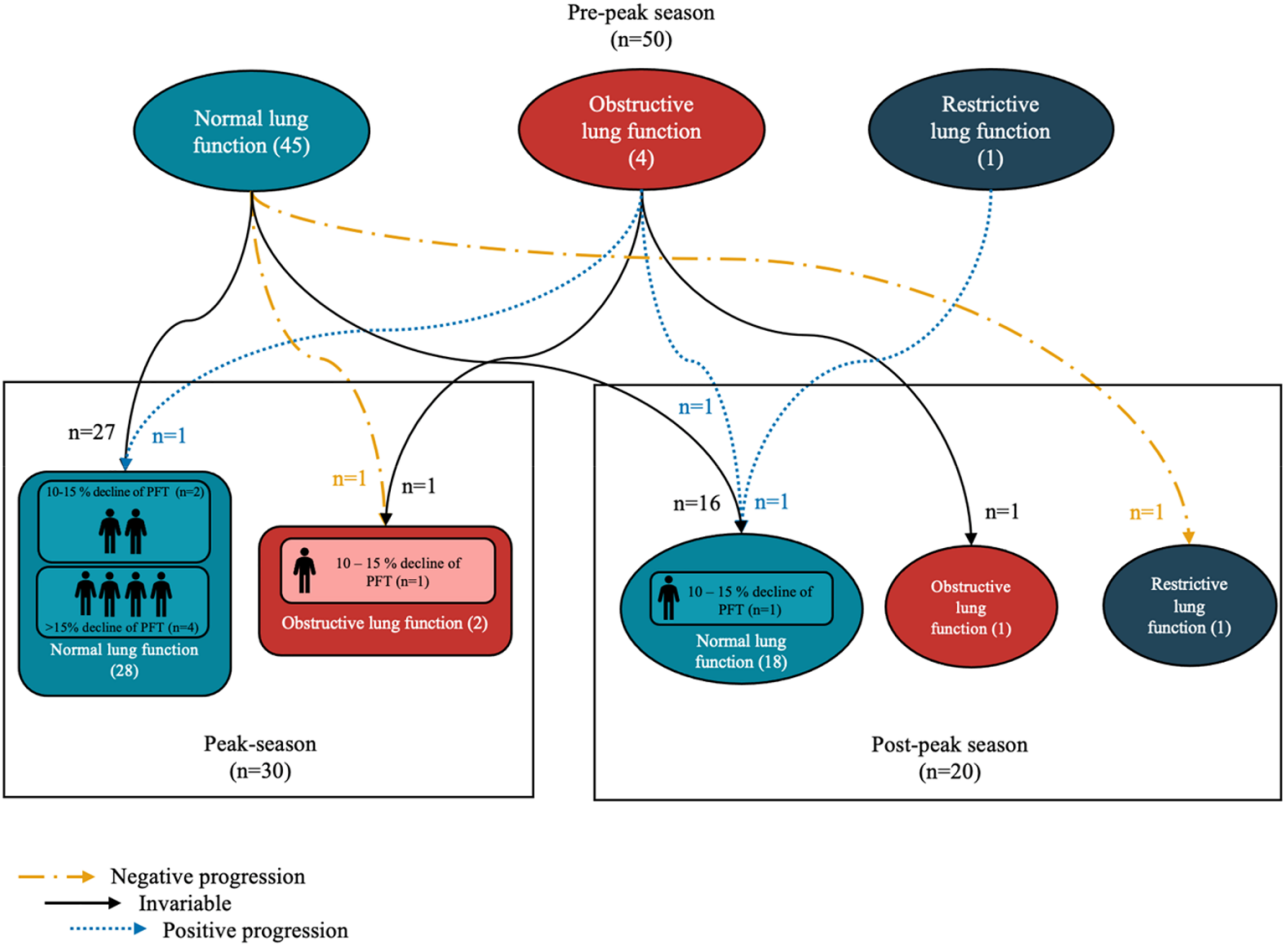
Classification of pulmonary function and its changes between pre-to-peak to post-season periods.

The conclusion of pulmonary function and how it changes over each phase was described in Fig. 3.

**Figure 4:**
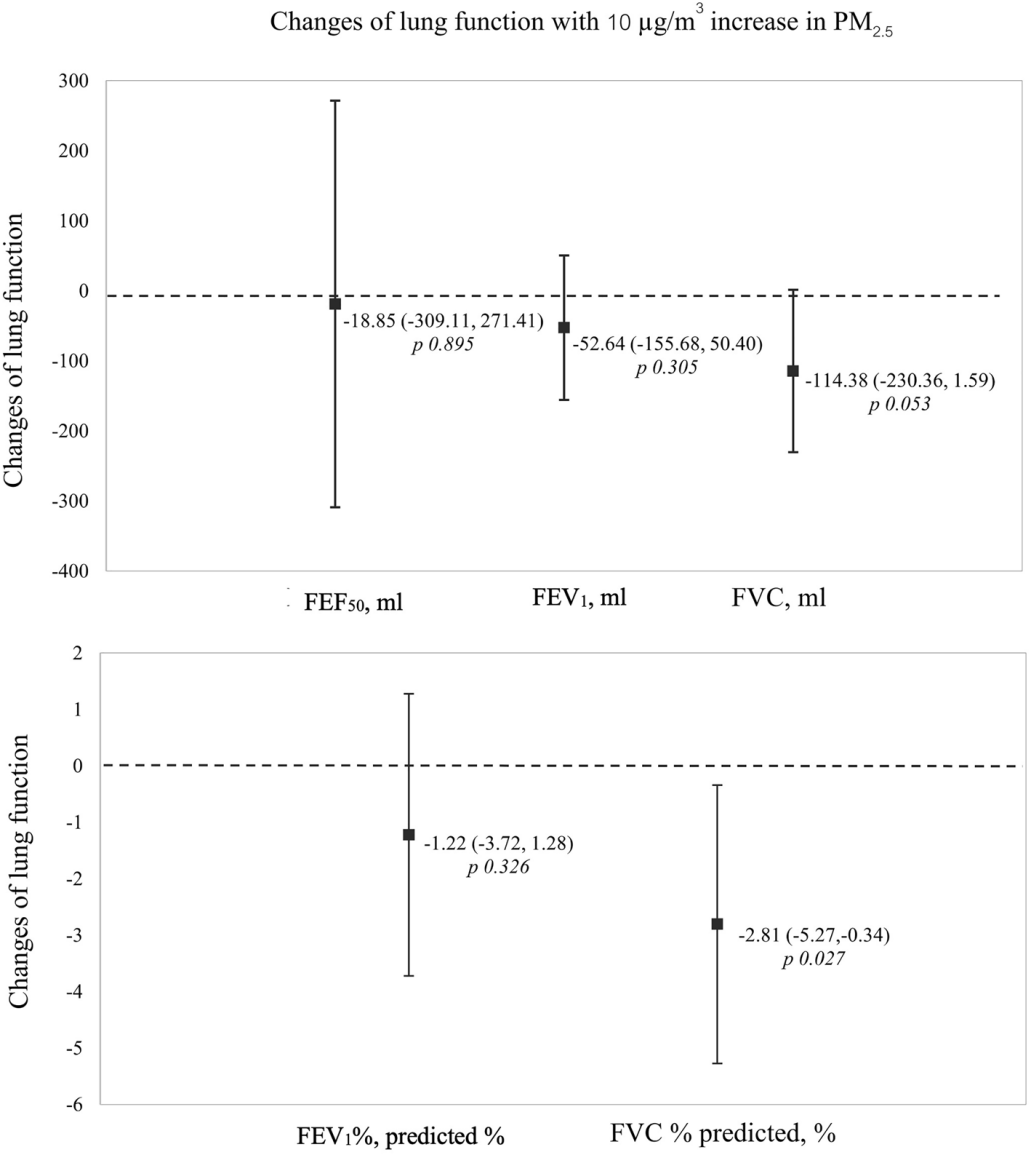
Multiple regression analysis of change in pulmonary function with 10 µg/m^3^ increase in PM_2.5_ adjusted by age, sex, smoking status, height, and weight. *FVC* Forced vital capacity, *FEF*_*50*_ Forced expiratory flow at 50% of the forced vital capacity, *FEV*_*1*_ Forced expiratory volume in 1 s, *L* liters.

Multiple regression analysis with adjustments for age, sex, smoking status, height, and weight in pre-peak group revealed a 10 g/m^3^ rise in PM_2.5_ had a negatively associated with the change in FVC% predicted (− 2.81%, 95% CI − 5.27 to − 0.34%, P = 0.027) and a marginally significant negative correlation with changing FVC (− 114.38 ml, 95% CI − 230.36 to 1.59 ml, P = 0.053). Other lung function parameters (FEV_1_, FEF_50_, and FEV_1_% predicted) showed negative direction without statistically significant. Change in pulmonary function with 10 µg/m^3^ increase in PM_2.5_ was showed in Fig. 4.

### DNA damage between two groups: tail length (TL) and tail moment (TM)

The mean change in the total case analysis (N = 49) was − 0.039 (95% CI − 0.230 to 0.152, P = 0.683) for TL and − 0.024 (95% CI − 0.010 to 0.051, P = 0.520) for TM. Multiple imputations of the average TL and TM changes were performed in 100 datasets using chained multivariate regression on an evaluation of the following baseline characteristics: age, gender, SBP, DBP, heart rate, BMI, TL, and TM. This method was applied to include an additional five right-censoring cases (10.2%) with presumed missing at random, as all missing participants came from a single forest fire control station. Results combined using Rubin’s rules were robust compared to the complete case analyses, with changes of − 0.037 (95% CI − 0.222 to 0.149, P = 0.691) for TL and − 0.023 (95% CI 0.097 to 0.05, P = 0.538) for TM. Table 4 presented the mean changes from the complete data analysis and multiple imputation analyses. TL and TM similarly showed no significant changes in the pre-to-peak and pre-to-post-peak season groups.

**Table 4:** Multivariate regression analysis and multiple imputation analysis of DNA length and DNA moment among pre-to-peak and pre-to-post-peak season period.

	DNA tail length		P-value	DNA tail moment		P-value
	Mean changes	95% CI		Mean changes	95% CI	
Total						
Complete case (n = 49)	− 0.039	− 0.230, 0.152	0.683	− 0.024	− 0.010, 0.051	0.520
Multiple imputation (n = 54)	− 0.037	− 0.222, 0.149	0.691	− 0.023	− 0.097, 0.051	0.538
Pre-to-peak season (January to March)						
Complete case (n = 28)	− 0.072	− 0.312, 0.168	0.546	− 0.039	− 0.138, 0.060	0.422
Multiple imputation (n = 33)	− 0.055	− 0.284, 0.174	0.626	− 0.032	− 0.131, 0.067	0.512
Pre-to-post-peak season January to May						
Complete case (n = 21)	0.004	− 0.330, 0.339	0.968	− 0.004	− 0.130, 0.122	0.920

Adjusted for age, gender, SBP, DBP, heart rate, and BMI.

## Discussion

Exposure to air pollution from wildfire smoke was a significant challenge for health among WLFF. This study highlighted the observed decline in pulmonary function during peak-pollution season among WLFF. The result found a 10 μg/m^3^ increase in PM_2.5_ was associated with declines in both the actual and predicted percentages of FVC and FEV_1_. However, it is worth noting that only the alteration in the predicted FVC percentage demonstrated statistical significance. The finding was reached along with the findings of several studies that demonstrated a decline in pulmonary function following exposure to wood and biomass combustion as PM_2.5_^7,8,24,25^. Some of the previous studies have demonstrated a dose–response relationship, whereby an increase of 10 μg/m^3^ in short-term and long-term exposure to PM_2.5_ is associated with reductions in FEV_1_ and FVC^24,26^. We observed mean reductions of 52.64 ml in FEV1 and 114.38 ml in FVC. Nevertheless, the volume change observed in this study was greater than that found in the US WLFF study^25^. The meta-analysis conducted in occupational studies revealed that an increase of 10 µg/m^3^ in PM_2.5_ was associated with a reduction in FVC of 10.0 ml (95% CI − 18.62 to − 1.37 ml) and FEV_1_ of 7.63 ml (95% CI − 10.62 to − 4.63 ml) for a 10 µg/m^3^ increase PM_2.5_^24^. In addition, the studies focused on WLFF showed the cross-shift changes in lung function, with a decline in FEV_1_ ranging from 30 to 50 ml^7,25^. Since previous studies primarily examined the immediate effects of occupational exposure after firefighting, it is plausible that repeated exposure could have a more significant impact on the number of declines observed during the study's pre-peak to peak season.

The pre-to-peak WLFF group exhibited a significant decline in FEV1 exceeding 10% from the baseline measurement, despite maintaining normal pulmonary function prior to and during exposure to air pollutants. Specifically, four out of six participants experienced a decline surpassing 15%. The outcome aligned with the conclusions presented by Gaughan DM. et al.^7^. The study involved US WLFF during the preseason, during-fire, and postseason of wildfires and revealed that 32.7% of US WLFF experienced a decline in FEV_1_ greater than 10% after a cross-shift, while only one participant demonstrated a decline in FEV_1_ exceeding 10% across the pre-to-post-peak season. Similar results were noted in studies involving firefighters and farmers exposed to wood and biomass burning^7,8,27^. These studies showed a decline in both FEV_1_ and FVC from the preseason to during-fire phase and an increase FEV_1_ and FVC from during-fire to postseason phase. However, even with the increase, the values remained lower than the baseline measurements. The timing of exposure, duration, lag time, and recovery time are also linked to the response of pulmonary functions. Short-term exposure can lead to cardiopulmonary effects with a lag time ranging from 0 to 7 days^28,29^. A washout period may play an essential role in the recovery of the pulmonary response to PM_2.5_. Observations indicate that cessation of exposure to high PM_2.5_ concentrations for 2–3 months improves pulmonary function^7,8,27^. In contrast, the study conducted by Jacquin et al. revealed a persistent reduction in FEV_1_ and FVC among WLFF even 3 months after end of fire season^8^. It is important to note that chronic exposure to particulate matter continues to have adverse effects on WLFF^26^. This suggests the potential lasting respiratory consequences can associated with the inhalation of forest fire smoke. Based on the evidence we have, we hypothesize that the WLFF in Chiang Mai was impacted on pulmonary function as a result of seasonal exposure to PM2.5 in the region. Even though ambient and indoor air pollution in this study did not differ significantly between pre-peak and peak seasons, there was still a trend of change in pulmonary function among WLFF without diagnosed abnormal spirometry. In addition, our findings suggested a partial improvement in the lung function with firefighting by the time we conducted our evaluation at the post-peak season. Therefore, further investigation on the recovery phase following the post-season to gather more informative data.

To gain further insight into the potential cause associated with the change in pulmonary function, we assessed the existence of DNA damage in WLFF. Several previous reviews indicate a negative association between particulate matter and DNA via mechanisms of inflammation and oxidative stress^12,30,31^. Surprisingly, this study found a non-statistical-significant change in DNA TL and DNA TM among pre-peak and pre-post-peak seasons. During the period of data collection, the concentrations of ambient and indoor particulate matter were observed to be lower compared to previous studies that reported significant changes in DNA damage markers^32,33^. Furthermore, several previous studies concentrated on short periods of exposure, in contrast to our extended monitoring duration^34,35^. This could be one of the causes of non-statistically significant changes and different DNA damage outcomes. Previously, there was a proposition suggesting that the comet assay might be useful as a suitable technique for monitoring occupational exposure. Nevertheless, the outcomes of its Evaluation of DNA-damaging Exposures in Occupational Environments were subject to controversy^36^. Comet assays might be more advantageous for assessing exposure than surveillance biomonitoring approaches^37^.

To our best knowledge, this study is a pioneering study trying to define the health outcomes for wildland firefighters in Thailand and find the tools to be used for proper surveillance. The study tries to assess more specific exposure assessment among WLFF in Thailand by simulating the daily activities of WLFF, including workplace indoor assessment, and comparing it with ambient air at the same time. It also tries to cover all periods of the annual season of pollution, including pre-peak and post-peak seasons. Nonetheless, this study had a few weaknesses that needed to be discussed. First, the duration of follow-up and assessment method may not represent the actual amount of exposure in cases of direct exposure after firefighting in expatriate areas. The event of a wildfire cannot be predicted, and we need to be concerned about interfering with WLFF during wildfire suppression. So, they were not available for us to follow them while wildfire events occurred due to our research team's safety. Second, the non-significant changes observed in certain pulmonary parameters and DNA damage during the peak season could potentially be attributed to the healthy worker effect and the limited sample size. WLFF requires a high level of physical exertion and optimal cardiopulmonary fitness to carry out strenuous job tasks, which may lead to increased physical training and self-care practices. The limited sample size might also have influenced the statistical power to detect significant differences in the measured parameters since a trend toward change in pulmonary parameters was observed.

The findings of this study indicate that Thai WLFF must establish a comprehensive medical surveillance system. We suggested implementing a spirometry pulmonary function test as an essential respiratory health monitoring tool for WLFF. In addition to interpreting spirometry results based on criteria for normal, obstructive, and restrictive abnormalities, it is essential to analyze a substantial decline of 10–15% in forced FEV_1_ compared with baseline spirometry measurements. It will be essential to implement this comprehensive approach is essential for early detection and optimal management strategies, which can be generalized to WLFF on a global scale. In addition, to reduce or minimize smoke exposure, it is recommended that WLFF implement a rotational work schedule involving the performance of other duties with lower levels of exposure. We also encourage the use of personal protective equipment to reduce smoke and PM_2.5_ exposure during fire suppression, evidence found that individuals who reported infrequent use of respiratory protection faced a greater risk of experiencing a faster decline in FEV_1_ compared to those who used it more frequently^38^. To strengthen the findings in future research, more participants are needed to increase the statistical power and result in more conclusive results. An accurate exposure assessment may be planned, and WLFF may be consulted to determine the proper approach for assessing actual exposure during the suppression of wildfires.

## Conclusion

The findings of this study concluded that Thai WLFF had a crucial need to be concerned about their health, either in terms of their fitness for work or their work-related health effects. Pulmonary function deterioration can manifest rapidly, yet there is a lack of evidence regarding the possibility of recovery. Spirometry was essential for identifying early changes in the pulmonary status of WLFF due to their work, which could impact their ability to perform specific job duties. It serves as a valuable tool for health surveillance. Additional research is required to understand the alterations in DNA damage and their potential association with PM2.5 exposure. Real-time exposure evaluation during the process of wildfire suppression may be necessary to make adjustments to an existing exposure assessment methodology.

### Supplementary Information

Supplementary Information.

## Data Availability

The data presented in this study are available from the corresponding author upon request.
