# Supplementary material for: Exploring the adverse effect of fine particulate matter (PM2.5) on wildland firefighters’ pulmonary function and DNA damage
Source: Sci Rep. 2024 Apr 4;14:7932. doi: 10.1038/s41598-024-58721-4 (PMC10994925; doi:10.1038/s41598-024-58721-4)
Supplement: Supplementary file 1 — Supplementary Information. [file 41598_2024_58721_MOESM1_ESM.docx]

**Exploring the Adverse Effect of Fine Particulate Matter (**PM_2.5_**) on Wildland Firefighters' Pulmonary Function and DNA Damage**

Jinjuta Panumasvivat^a,b^, Ratana Supbamrer^a,b^, Nalin Sittitoon^c^, Supakit Khacha-ananda^d^, Wuttipat Kiratipaisarl^a^, Wachiranun Sirikul^a,b,e^, Wittawat Insian^f^, Pheerasak Assavanopakun^a,b^*

^a^ Department of Community Medicine, Faculty of Medicine, Chiang Mai University, Chiang Mai, 50200, Thailand

^b^ Occupational and Environmental Medicine Unit, Department of Community Medicine, Faculty of Medicine, Chiang Mai University, Chiang Mai 50200, Thailand

^c^ School of Environmental Health, Institute of Public Health, Suranaree University of Technology, Nakhon Ratchasima, 30000, Thailand

^d^ Department of Forensic Medicine, Faculty of Medicine, Chiang Mai University, Chiang Mai, 50200, Thailand

^e^ Center of Data Analytics and Knowledge Synthesis for Health Care, Chiang Mai University, Chiang Mai, 50200, Thailand.

^f^ Office of Disease Prevention and Control 1 Chiang Mai, Department of Disease Control, Ministry of Public Health, Chiang Mai, 50000, Thailand

* **Corresponding Author:** Pheerasak Assavanopakun, MD, MSc

Community Medicine, Faculty of Medicine, Chiang Mai University, 50200, Thailand

Tel: +6653-935472

E-mail: Pheerasak.assava@cmu.ac.th

DNA damage was measured using the alkali comet assay based on the method published by Singh et al. (18).

**Step 1: Slide preparation were as follows:**

Slide was immersed overnight in 70% Methanol, and then coated with 1% normal melting point agarose.

**Step 2: Sample preparation were as follows:**

1. Ten milliliters of blood sample was collected from individual subjects in blood collection tubes containing heparin.
2. Blood sample was transferred to be centrifuged at 3000 revolutions per minute (rpm) for fifteen minutes to enable the collection of plasma.
3. Plasma sample was diluted with 1 mL of normal saline, and then centrifuge at 1,000 rpm for 30 minutes at 4 ^0^c to enable the collection of lymphocytes.
4. 20 μL of lymphocytes were mixed with 75 μL of Low melting point agarose, and placed the cell on a slide.
5. The slide with sample was immersed in lysing solution (1% sodium sarconisate, 2.5 M NaCl, 100 mM Na2EDTA, 10 mM Tris-HCl, 10% DMSO and 1% Triton X-100) at 4 ^o^c for 1 hr., and then immersed in alkaline buffer (0.3 M NaOH, 1 mM Na2EDTA, pH 13) for 10 minutes.

**Step 3: Electro electrophoresis (running the gel) were as follows:**

1. After the cells were lysed, DNA was denatured and run through electrophoresis.
2. The slide with sample was treated with alkaline buffer at condition of 25 volts, 300 milliamps, and 4^o^c for 20 minutes, and then immersed in neutralized buffer for 10 minutes.
3. The slide with sample was dyed with 10% ethidium bromide at concentrations of 20 μg/mL, and then covered with slide cover.
4. The slide was kept at 4 ^o^c until analysis.

**Step 4: Visualization and documentation were as follows:**

1. The slide with sample was analyzed by using a fluorescence microscope equipped with an imaging system and COMET Assay software.
2. The resulting image of tail length (TL) and tail moment (TM) of DNA were measured.

TL = distance of DNA migration

TM = (Distance between the center of gravity of the head to the center of gravity of the tail) X (Tail DNA Intensity/Total Comet DNA intensity)
